# Self-supported nickel nanoparticles on germanophosphate glasses: synthesis and applications in catalysis

**DOI:** 10.1039/c9ra02927c

**Published:** 2019-05-31

**Authors:** Guilherme Felipe Lenz, Rodrigo Schneider, Kelen M. F. Rossi de Aguiar, Rafael A. Bini, Juliano Alexandre Chaker, Peter Hammer, Giancarlo V. Botteselle, Jorlandio F. Felix, Ricardo Schneider

**Affiliations:** Universidade Federal do Paraná - UFPR, Departamento de Engenharias e Exatas Palotina PR 85950-000 Brazil; Universidade Tecnológica Federal Paraná - UTFPR, Group of Polymers and Nanostructures Toledo PR 85902-490 Brazil +55 45 33796850; Universidade Federal de São Carlos, Departamento de Química São Carlos SP 13565-905 Brazil; Universidade de Brasília - UNB, Instituto de Química Brasília DF 70904-970 Brazil; Universidade do Estado de São Paulo - UNESP, Instituto de Química Araraquara SP 14800-060 Brazil; Universidade Estadual do Oeste do Paraná - UNIOESTE, Departamento de Química Toledo PR 85903-000 Brazil; Universidade de Brasília - UNB, Instituto de Física, Núcleo de Física Aplicada Brasília DF 70910-900 Brazil jorlandio@unb.br

## Abstract

The development of supported catalysts based on simple procedures without waste products and time-consuming steps is highly desirable. In this paper, self-supported nickel-based nanoparticles were obtained at the surface of the germanophosphate glasses by bottom-up process and evaluated as potential catalysts for the benzyl alcohol oxidation and bis(indolyl)methanes synthesis. A classical melt-quenching technique was used for preparing the nickel-doped germanophosphate glasses, followed by annealing under a hydrogen atmosphere at 400 °C for two different times. The approach enabled the synthesis of self-supported nanoparticles as a homogeneous film, covering the glass surface. The physical and chemical properties of synthesized glasses were characterized by UV-vis and Raman spectroscopies and thermal analysis. Scanning electron microscopy (SEM) and X-ray photoelectron spectroscopy (XPS) were performed to monitor the growth process, morphology and chemical bonding structure of the nanoparticles surface.

## Abstract 2

The development of supported catalysts based on simple procedures without waste products and time-consuming steps is highly desirable.

## Graphical abstract

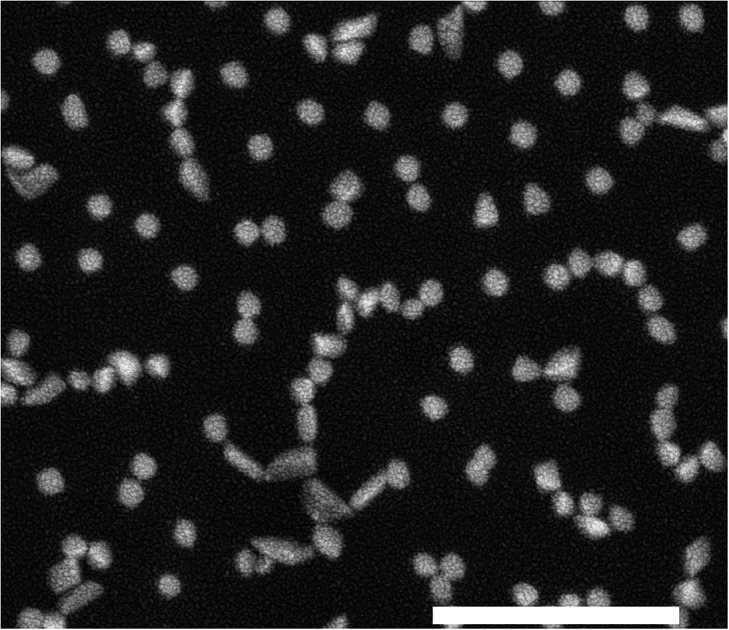

## Introduction

Catalysis presents a significant role in industrial processes. Catalytic materials/nanomaterials show applications in chemical manufacturing, energy, biological and environmental fields.^1,2^ The outstanding properties of nanocatalysts are associated with high catalytic activity and easy separation. Nanoparticles (NPs) from noble metals are extensively studied for organic transformations and applied in pharmaceutical process, for example.^3^

Nanoparticles can be synthesized by a variety of conventional methods using gas-, liquid-, or solid-phase processes. These methods include some undesirable variables such as gas-phase reactions of flame pyrolysis, high-temperature evaporation, plasma, microwave irradiation, *etc.*^4^ In addition, colloidal or liquid-phase methods are also employed, chemical reactions in solvents lead to the formation of colloidal solutions. Other methods involve solvent-based colloidal or liquid-phase synthesis, molecular self-assembly and mechanical processes of size reduction such as grinning, milling, and alloying.^3,4^

Therefore, the development of a simple and efficient approach for the synthesis of immobilized nanoparticles is desirable. In this sense, over the last few years, our research group has developed a non-conventional route for the growth of copper and silver nanoparticles on borophosphate, oxide and oxyfluoride glasses. We have also evaluated their performance in catalysis, Surface-enhanced Raman Spectroscopy (SERS), and antibacterial applications.^5–9^

Phosphate-based glasses can be obtained at relatively low temperature by melt-quenching technique using affordable chemical reagents (*e.g.*, KH_2_PO_4_, P_2_O_5_, NaPO_3_ or NaH_2_PO_4_). In addition, this type of glass becomes even more interesting because it can be easily doped with transition metal ions, alkali, and rare earth oxides to provide the desired physical and/or chemical characteristics.^10^ Moreover, phosphate glasses can act as a host material for nanoparticle synthesis enabling the growth of nanoparticles onto glass surface when annealed under a reductive atmosphere (*e.g.*, hydrogen gas). In general, silver, gold, and copper are the transition metals more reported which can be precipitated inside glasses by chemical reactions, annealing or radiation exposure.^11–13^ So *et al.*^14^ reported the synthesis of PbS quantum dots in glasses containing silver nanoparticles using laser illumination, while Estournès *et al.*^15,16^ reported the precipitation of nickel nanoparticles in soda-lime-silicate glass, when annealing under hydrogen atmosphere at 600 °C. Their results showed that the annealing process produced nickel nanoparticles embedded in silica-based glass. In terms of catalytic applications, the nanoparticles should be accessible for the reactions and, therefore, embedded nanoparticles (*i.e.* surrounded by support) are undesirable. Nickel-based catalysts are applied for heterogeneous catalysis with practical applications in the industry (*e.g.*, methanation reaction, oxidation of carbon monoxide, hydrogen-transfer reductions).^17^

In this work, we report the synthesis and application of germanophosphate glass, which acts as a host and a support material for the growth of nickel nanostructures onto its surface (not embedded in glass matrix). The self-supported nickel-based nanoparticles were obtained at relatively low temperature and short annealing time in form of a homogeneous layer on the glass surface. Then, the supported nanoparticles were applied as catalyst in benzyl alcohol oxidation by sodium hypochlorite and in Friedel–Crafts alkylation reaction of indole and benzaldehyde. The structural characterization was performed using a combination of techniques such as differential thermal analysis, UV-vis spectroscopy, X-ray photoemission spectroscopy, scanning electron microscopy and Raman spectroscopy.

## Experimental

### Glass synthesis and Ni-based nanoparticles growth

Germanophosphate glasses (NaH_2_PO_4_–GeO_2_–Al_2_O_3_) were obtained by melting-quenching technique using high purity reagents (Aldrich Co.) with NaH_2_PO_4_/GeO_2_ = 2 and 3 mol% of Al_2_O_3_. The nickel-doped glass was prepared with addition of 1.5 mol% Ni_2_O_3_ (3 mol% Ni^2+^ ions). A typical synthesis was performed by homogenizing 2 g of the aforementioned compounds in an Agate mortar. The mixture was transferred to a covered Pt/Au crucible and heated at 950 °C for 1 h, in a preheated resistive oven. Afterwards, the molten sample was quenched at room temperature in a graphite mold. Finally, the glasses were crushed in an agate mortar and sieved on a 325-mesh sieve. Nickel-based nanoparticles were obtained by annealing the nickel-doped glasses at 400 °C for 30 and 60 minutes under hydrogen (H_2_(g)) flow of 100 mL min^−1^.

### Glass and nanoparticles characterization

The characteristic glass temperatures of the samples were determined by Differential Thermal Analysis (DTA-50, Shimadzu) using the powdered glass (325 mesh) in a Pt pan with nitrogen gas flow (50 mL min^−1^) and heating rate of 10 °C min^−1^.

The local bonding structure of the samples was evaluated by X-ray photoelectron spectroscopy (XPS) using a commercial spectrometer UNI-SPECS UHV, at a base pressure of 5 × 10^−7^ Pa. Al K_α_ radiation source was used (*hν* = 1486.6 eV) and the pass energy of the analyzer was adjusted to 10 eV. The inelastic noise of the high resolution spectra of Ni 2p_3/2_, Ge 3p, Na 1s, P 2p, and O 1s were subtracted using the Shirley method.^18,19^ The composition (at%) of the surface layer (<5 nm) was determined by the relative proportions of peak areas corrected for Scofield's atomic sensitivity factors to an accuracy of ±5%.^20^ The binding energy calibration was performed by referring the C 1s component of aliphatic carbon species to 285.0 eV. The spectra were deconvoluted using a Voigtian type function, with combinations of 70% Gaussian and 30% Lorentzian. The width at half maximum varied between 1.1 and 2.0 eV, and the position of the peaks was determined with an accuracy of ±0.1 eV.

The Raman spectra were recorded using a micro-Raman Renishaw InVia, laser power 8 mW, 633 nm excitation wavelength and CCD detector. All samples were measured without any additional treatment. The deconvolution analysis of Raman spectra was obtained with Voigt functions using Fityk program (version 1.3.1). The UV-vis absorption spectra of glasses (≈0.5 mm thick foils) were recorded in the 200–1000 nm range using a T80+ spectrometer from PG instruments with 1 nm of step and air as baseline.

### Catalytic measurements for benzyl alcohol oxidation

Nickel-doped germanophosphate glass was annealed at 400 °C during 60 minutes under H_2_ atmosphere (100 mL min^−1^) and tested as catalyst for benzyl alcohol (BnOH) oxidation by sodium hypochlorite (NaOCl) solution. The sample annealed during 60 minutes was chosen for the catalytic tests due to its larger number of nickel nanoparticles at the glass surface, as will be shown later in the SEM results (Fig. 4). The sample obtained with annealing time of 60 minutes was chosen for the catalytic tests due to its larger number of nickel nanoparticles at the glass surface, as will be shown later from SEM results.

The catalytic evaluation was initially performed in aqueous media using NaOCl 5 wt% as oxidant solvent at pH 10 (10 mL, 6.7 mmol). 100 mg of the catalyst was first dispersed in the NaOCl solution, followed by the addition of 1.50 mmol of BnOH. The reaction was performed at 20.0 °C ± 0.5 °C during 1 h. Posteriorly, the reaction condition was adapted by using acetonitrile (ACN) as solvent (10 mL) and sodium hypochlorite (NaOCl) 10–12 wt% applied only as oxidant (3 mL, 4.8 mmol). 100 mg of the catalyst was first dispersed in ACN, followed by the addition of 0.75 mmol of BnOH. Oxidant was added in portions of 1 mL at the beginning and within the first and second hour of reaction. The reaction was performed at 20.0 °C ± 0.5 °C during 8 h.

The unconverted benzyl alcohol (BnOH) and the products benzaldehyde (BnCHO), benzoic acid (BnCOOH) and benzyl benzoate (BnBz) were determined using high performance liquid chromatography (HPLC) in a Thermo Scientific Ultimate 3000 equipment. The separation was performed at 30 °C using octadecylsilane C18 column (Ace ltd.) in gradient elution at a flow rate 1 mL min^−1^, with mobile phase composed by the mixture of acidified water (0.01% v/v phosphoric acid, pH 2.75 ± 0.05) and acetonitrile (ACN, J.T. Baker HPLC grade): initially 30% ACN was increase to 60% after 10 min., keeping this condition until 25 minutes. The detection was made by a diode-array detector at 210 nm. Aliquots of the reaction medium were collected at the beginning, during, and at the end of the reaction, and then diluted 10 times with mobile phase and filtered in a 0.22 μm hydrophilic PVDF syringe filter. Analytical standards (Sigma Aldrich, Supelco) were used as reference for sample concentration determination. To verify the proportion of compounds (mmols) in the formed phases, the organic reaction media was centrifuged (3400 rpm, 6 minutes) and a fraction of both organic and aqueous phases was collected for analysis.

### Synthesis of bis(indolyl)methane (3)

For synthesis of bis(indolyl) methane the Ni-based nanoparticles (25 mg), indole (0.50 mmol, 58.5 mg) and benzaldehyde (0.25 mmol, 26.5 mg) were added into a tube test (5 mL). The tube was then immersed in an oil-bath at 60 °C and stirred during 1.5 h. The organic compounds were then directly extracted with ethyl acetate (3 × 3 mL), dried over Na_2_SO_4_ and concentrated under vacuum. The crude product was purified by flash column chromatography on silica gel using a mixture of hexane and ethyl acetate (85 : 15) as the eluent. The identification of the product was confirmed by ^1^H and ^13^C nuclear magnetic resonance (NMR) according to the following data. 3,3′-(Phenylmethylene)bis(1*H*-indole) (3) (Section Glass catalyst performance): ^1^H NMR (CDCl_3_, 200 MHz): *δ* = 7.67 (br, 2H); 7.37–6.93 (m, 13H); 6.51 (s, 2H); 5.84 (s, ^1^H); ^13^C NMR (CDCl_3_, 50 MHz): *δ* = 144.3, 137.0, 129.0, 128.5, 127.4, 126.3, 123.8, 122.1, 120.1, 119.9, 119.4, 111.2, 40.3.

## Results and discussion

### Glass characterization

**Fig. 1:**
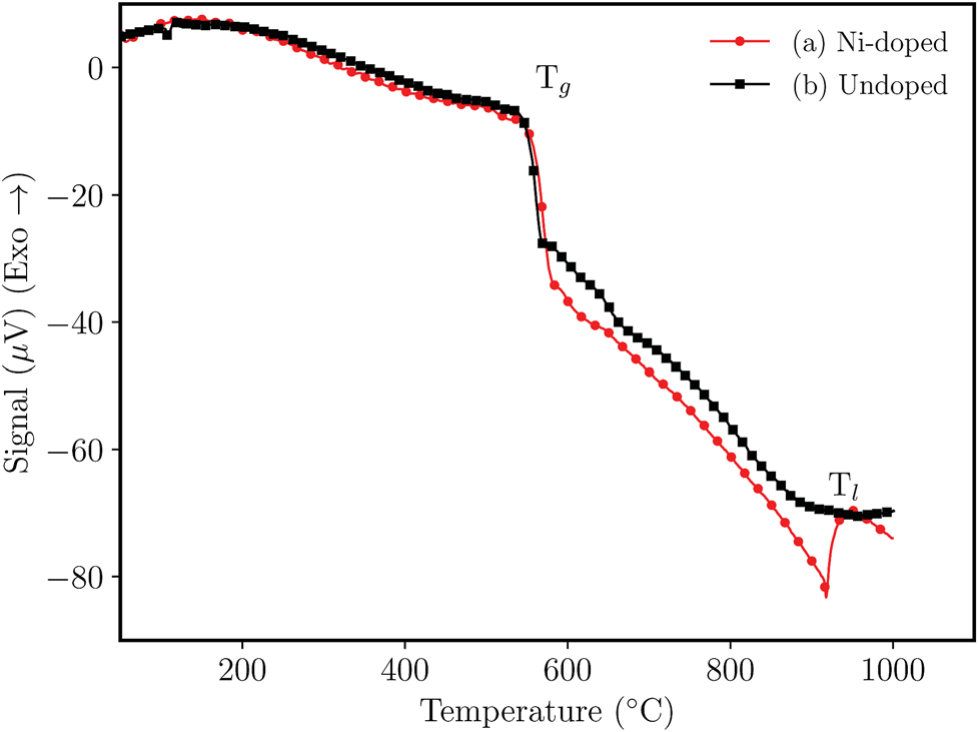
Differential thermal analysis (DTA) of glasses without annealing (a) 3 mol% Ni-doped, and (b) undoped sample.

Fig. 1 shows the DTA analysis of the NaH_2_PO_4_–GeO_2_–Al_2_O_3_ germanophosphate glasses. The glass transition temperature (*T*_g_) was noticed at ≈550 °C for the undoped sample (Fig. 1(b)) and 558 °C for Ni-doped sample (Fig. 1(a)). The liquidus temperature (*T*_l_) of the doped glass was noticed at ≈942 °C, while for the undoped glass the *T*_l_ was not observed within the analysis range. The addition of nickel ions increased the *T*_g_ indicating that the metallic ions inside the glass matrix act as a network intermediate.^21,22^

**Fig. 2:**
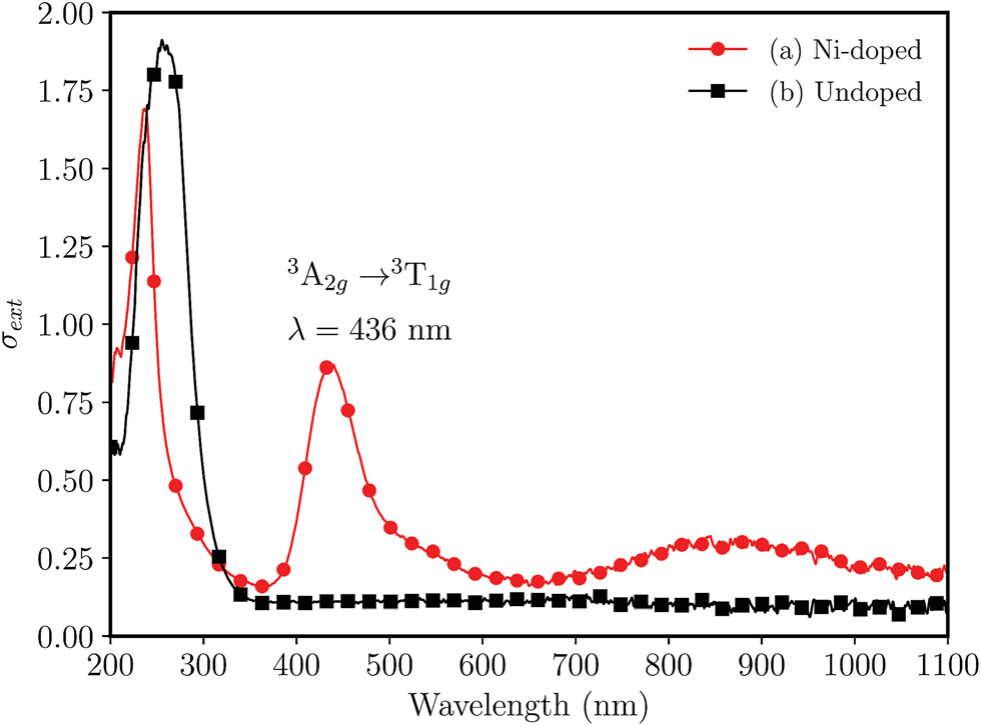
UV-vis absorption spectra for bulk (foils with ≈0.5 mm of thickness) 3 mol% Al_2_O_3_ germanophosphate glasses without annealing (a) 3 mol% Ni-doped, and (b) undoped.

The UV-vis absorption spectra for undoped and Ni-doped samples are shown in the Fig. 2. The undoped glass was colorless, (Fig. 2(b)) transmitting the radiation above ≈340 nm, while the Ni-doped germanophosphate glass shows an amber-colored aspect, evidencing the doping process. The band observed at 436 nm (Fig. 2(a)) was assigned to a spin-allowed transition ^3^A_2g_(F) → ^3^T_1g_(P) of Ni^2+^ ion in octahedral coordination.^23,24^

**Fig. 3:**
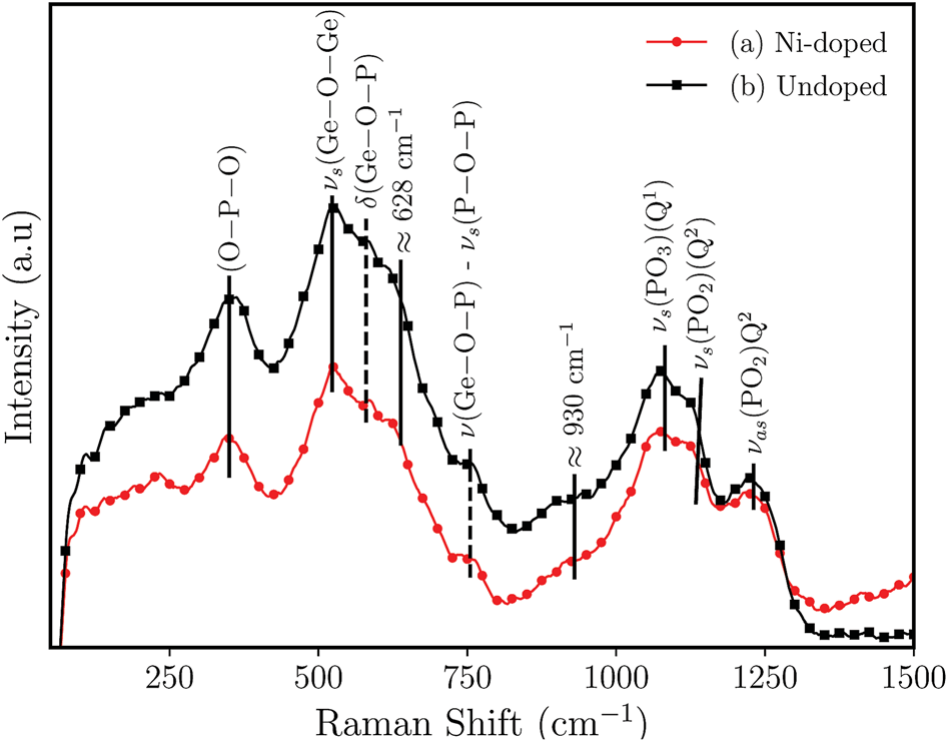
Raman spectrum for germanophosphate 3 mol% Al_2_O_3_ germanophosphate glasses without annealing (a) 3 mol% Ni-doped, and (b) undoped.

Fig. 3 shows the results of the Raman analysis of the synthesized germanophosphate glass samples. The Raman spectrum is mainly composed of two complex broad bands located below and above of ≈810 cm^−1^. The band located below 810 cm^−1^ is an overlapping of phosphate- and germanium-based groups. The band at ≈350 cm^−1^ is attributed only to O–P–O bending.^25,26^ The band at ≈523 cm^−1^ is assigned to symmetric stretching (*ν*_s_) of three-membered GeO_4_ rings.^26^ The peaks at 582 cm^−1^ and 757 cm^−1^ (the dashed lines (Fig. 3)) are attributed to *δ*(Ge–O–P) bending vibrations and *ν*(Ge–O–P) and/or *ν*_s_(P–O–P) (Q^1^ units) stretching frequencies, respectively.^26–29^ In turn, Kamitsos *et al.*^30^ ascribed the peak/shoulder at ≈628 cm^−1^ to the formation of connected GeO_6_ octahedral units. Alternatively, Kumar *et al.*^27^ ascribed the shoulder at ≈643 cm^−1^ to Ge_6_–O–P bending modes in Li_2_O–GeO_2_–P_2_O_5_ (P/Ge = 1.6) glasses. The region above 810 cm^−1^ can be deconvoluted with a series of four Voigts line shapes. The broad band is composed of peaks at ≈930, 1075, 1135 and 1230 cm^−1^. The peaks at 1135 cm^−1^ and 1230 cm^−1^ are assigned to symmetric *ν*_s_(PO_2_) (Q^2^), and to the asymmetric *ν*_as_(PO_2_) (Q^2^) stretchings.^31^ The peak at 1075 cm^−1^ is ascribed to *ν*_as_(PO_3_) in pyrophosphates (Q^1^).^26,29,32^ At last, the shoulder at ≈930 cm^−1^ is associated with *ν*_as_(PO_4_^3−^) (Q^0^) isolated orthophosphate units and to asymmetric stretching of *ν*_as_(P–O–P).^32–34^

The main advantage of the germanophosphate glass is the ability of nickel-based nanoparticles to grow on its surface when annealed at relatively low temperature. Basically, the annealing under hydrogen atmosphere enables the reduction of the nickel ions, thus inducing the growth of nickel nanoparticles at the glass surface, without additional steps. The mobility of nickel ions in the glass matrix increases with temperature rise, which favors their migration from the bulk to the glass surface for sequential reaction with the hydrogen atoms.

**Fig. 4:**
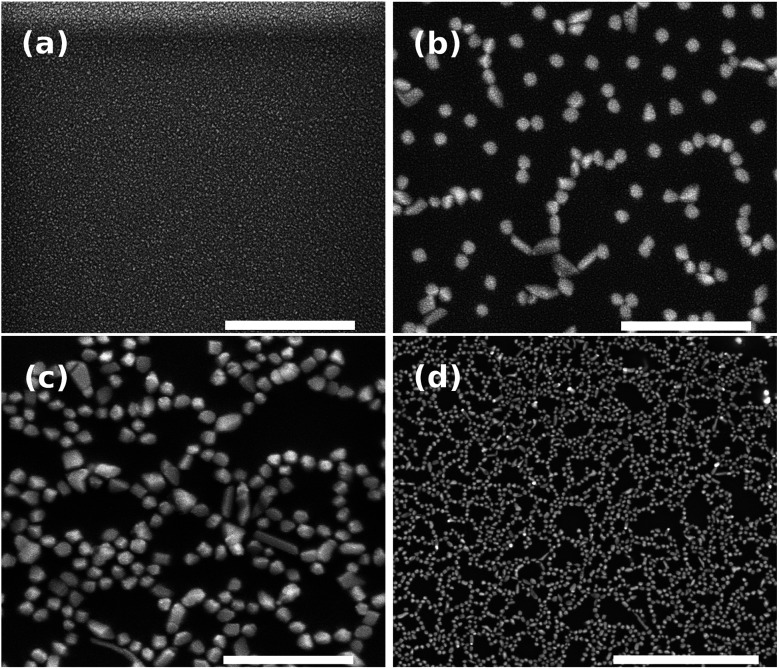
SEM images of powder Ni-doped germanophosphate glasses after different annealing times at 400 °C under H_2_ atmosphere of (100 mL min^−1^) (a) unannealed (scale bar = 1 μm), (b) 30 min (scale bar = 1 μm), (c) 60 min (scale bar = 1 μm) and (d) large area – 60 min (scale bar = 4 μm).

Fig. 4 shows the SEM images of nanostructures obtained on the glass surface after annealing. As expected, for the sample unannealed, we did not notice the nanoparticle (Fig. 4(a)) since the bottom-up process is thermally activated. It is worth mentioning that the nickel-based nanostructures growth requires a reductive atmosphere (*i.e.* H_2_(g)). In contrast to germanophosphate matrix, the growth of silver nanostructures can be carried out in lead-germanate glasses under a nitrogen atmosphere.^8^ On the other hand, the negative point of lead-germanate glasses is that they have heavy-metal in their matrix. Fig. 4(b) and (c) show the effect of the annealing time in the nanoparticle growth. For an isothermal process, the amount of nanoparticles on the glass surface increases with the annealing time. Moreover, the nanostructures can be observed covering homogeneously the glass surface (Fig. 4(d)).

**Fig. 5:**
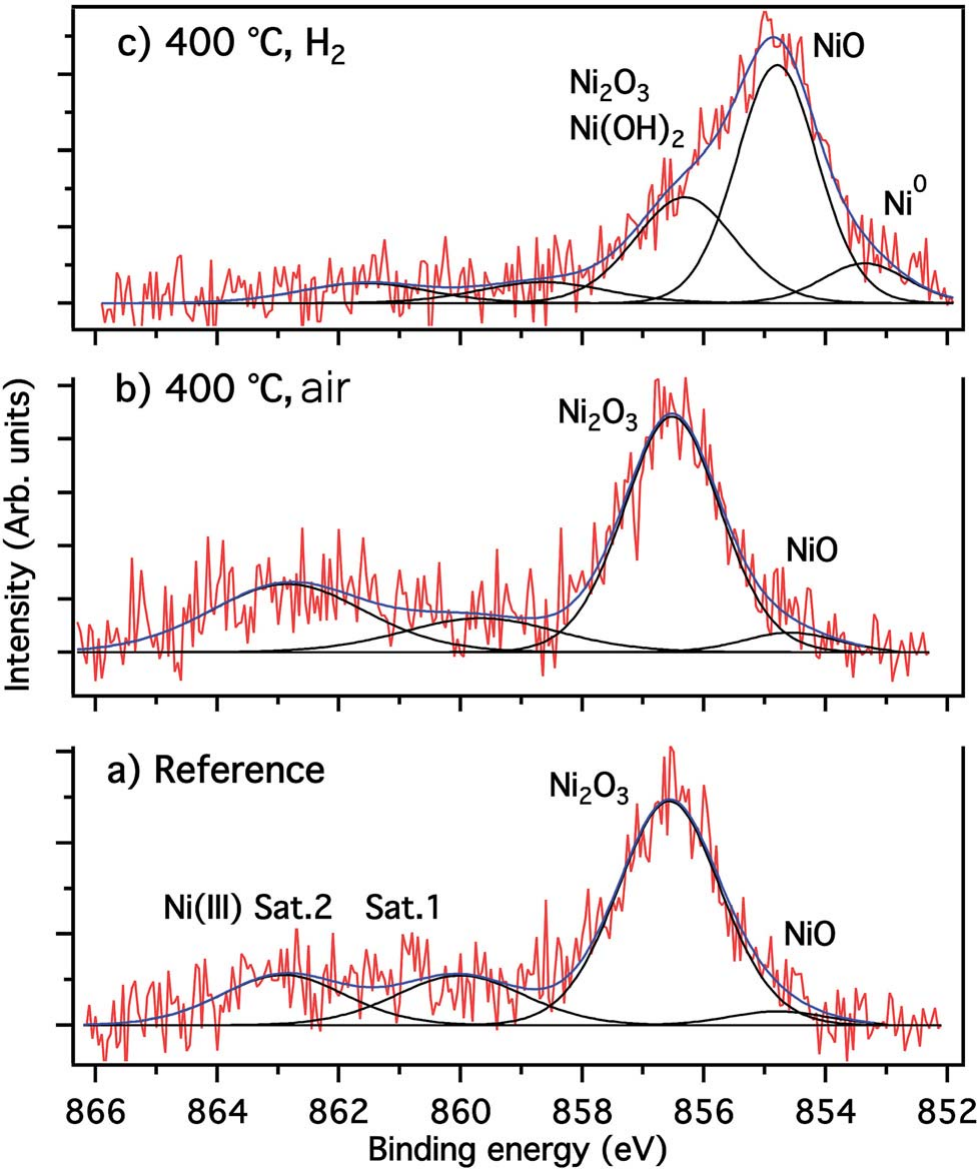
Fitted high resolution XPS Ni 2p_3/2_ spectra (a) reference (unannealed), (b) annealed under air, (c) annealed under H_2_.

To win an insight into the nanoparticle growth onto the glass surface, XPS measurements have been performed. The attribution of the fitted component and the corresponding fitting parameters obtained from XPS results are summarized in Table 1. Annealing in air was also performed to evince the beneficial effect of hydrogen for nanoparticle growth. The fitting of the spectrum of Fig. 5(a) and (b), was performed using four components. Only a small contribution (<4.5%) of the NiO phase was found at 854.6 eV, while the predominant Ni_2_O_3_ phase at 586.5 eV contributes with about 60%. Moreover, there are two shake up satellite relative to NiO (Ni II) at about 560 eV, and Ni_2_O_3_ (Ni III) at 563 eV.

**Table 1:** XPS fitting parameters for nickel-doped glass samples

Component	Reference^a^		400 °C, air^b^		400 °C, H_2_^c^	
	Position	Intensity (%)	Position	Intensity (%)	Position	Intensity (%)
**Ni 2p**_**3/2**_						
Ni^0^	—	—	—	—	853.4	9.8
NiO	854.6	4.48	854.6	3.90	854.3	49.1
Ni_2_O_3_	856.5	56.39	856.6	62.40	856.3	29.2
Ni(iii) Sat.1	859.7	12.99	860.0	17.70	860.1	8.2
Ni(iii) Sat.2	862.9	26.14	862.9	16.00	863.0	3.3

**O 1s**						
NiO	529.4	1.9	529.3	1.8	529.4	1.4
PO_4_, Ni_2_O_3_, Ni(OH)_3_	531.5	60.3	531.5	56.5	531.4	59.2
GeO_2_, O–C	532.7	20.4	532.6	23.5	532.6	19.8
O–C=O	533.8	4.8	533.7	4.6	533.7	3.8

a Fig. 5(a).

b Fig. 5(b).

c Fig. 5(c).

The Ni 2p_3/2_ spectrum of Fig. 5(c) shows clearly that the sample annealing under H_2_ favors the reduction of nickel species when compared to the reference sample (Fig. 5(a)) and the sample annealed under air (Fig. 5(b)). Besides the formation of approximately 10% of metallic nickel, the reductive H_2_ atmosphere results in a strong increase of the amount of NiO by up to 50%, and a reduction of Ni_2_O_3_ species (Table 1 and Fig. 5(c)). Furthermore, the small shift of the latter component from 856.6 eV to 856.3 eV indicates at this binding energy the presence of Ni(ii) species in form of Ni(OH)_2_, as revealed by the weak intensity of the Ni(iii) shake-up and the analysis of the O 1s spectra (not shown).

The results of the fitted O 1s, shown in Table 1 confirmed the presence of the NiO phase at 529.4 eV, PO_4_, Ni_2_O_3_ and Ni(OH)_2_ at 531.5 eV as well as O–Ge and O–C bonds at 532.6 eV.^35^ The O–C and carboxyl groups (O–C=O), identified at 533.7 eV, can be related to a weak contamination of the sample surface by adventitious carbon (hydrocarbons). Moreover, the P 2p_3/2_/2p_1/2_ spin–orbit spectra of phosphorus confirmed the presence of the PO_4_ phase at 133.6 eV, and the position of the Na 2s spectra of the sodium at 63.6 eV is indicative for the NaH_2_PO_4_ phase (not shown).

### Glass catalyst performance: benzyl alcohol oxidation

**Fig. 6:**
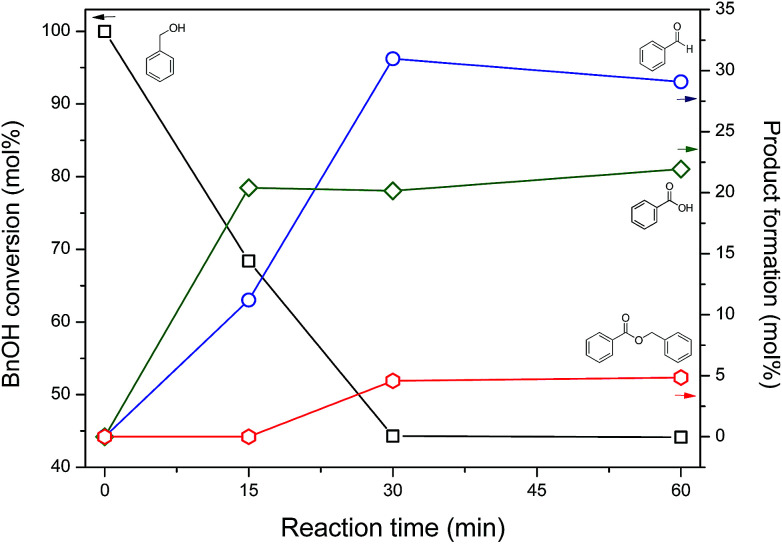
Benzyl alcohol conversion at 20 °C under agitation, and obtained products in aqueous NaOCl 5% pH 10 using self-supported nickel-based nanoparticles as catalyst.

Fig. 6 shows benzyl alcohol (BnOH) conversion and product yields, according to the reaction time, in aqueous NaOCl 5% pH 10, using as catalyst nickel-based nanoparticles obtained after 60 minutes of annealing: BnOH conversion achieved 55.7 mol% in 30 minutes, maintaining it constant until 60 minutes. Benzaldehyde (BnCHO) was the major product of reaction – 29 mol% at 60 minutes, while benzoic acid yield reached 22 mol%. The benzyl benzoate, ≈5 mol%, was noticed as product of esterification between benzyl alcohol and benzoic acid. The catalytic activity and effectiveness of the nickel-based nanoparticles were evinced testing the reaction without the catalyst, where only 2.6 mol% of conversion was achieved after 60 min reaction.

Sodium and calcium hypochlorite are known as strong oxidants, being able to convert primary alcohols to its respective esters, secondary alcohols to ketones and aldehydes to acids/esters.^36–38^ Nevertheless, commercial NaOCl solution (bleach) was used with a nickel(ii) salt to oxidize benzyl alcohol to benzoic acid, as reported by Grill and co-workers.^39^ The authors reported benzaldehyde as an intermediary of the reaction, easily oxidized to acid. However, applying our reaction conditions, benzaldehyde was the major product instead of benzoic acid, achieving 51% of selectivity to the aldehyde.

**Fig. 7:**
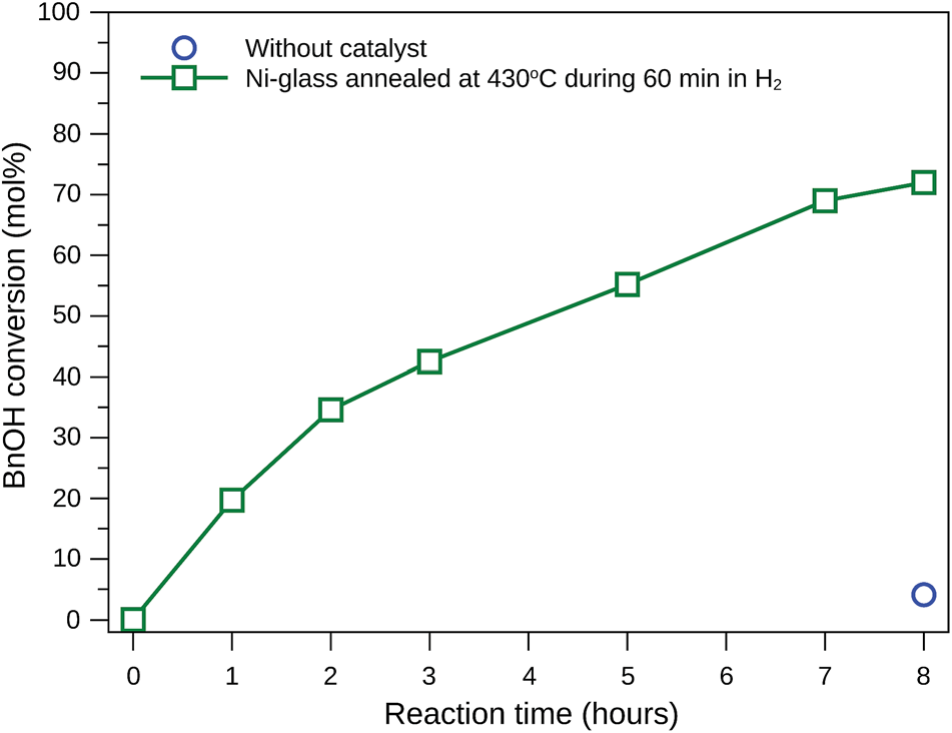
Benzyl alcohol conversion at 20 °C using acetonitrile as solvent, NaOCl as oxidant (4.8 mmol) and self-supported nickel-based nanoparticles as catalyst.

Many researchers have tried to find reactions conditions and catalysts that favor benzaldehyde production, mainly due to its extensive industrial application.^40–42^ Therefore, this study focuses on the test of glass-based catalyst and reaction conditions that provide selectivity to benzaldehyde. Fig. 7 shows BnOH conversion when acetonitrile (ACN) was used as solvent and NaOCl 10–12% as oxidant, with the reaction being conducted at 20 °C under agitation. The BnOH conversion achieved 72 mol% after 8 hours of reaction when the catalyst was grown during 60 minutes, while only 5.7 mol% of conversion was obtained without the catalyst.

The BnOH oxidation, carried out in aqueous medium, lead to the formation of three products (mixture): benzaldehyde, benzoic acid and benzyl benzoate. Water favours the oxidation of BnCHO due to the hydration, generating BnCOOH,^43^ and the basic pH (10.0) allows the esterification (*i.e.* generating BnBz). Nevertheless, the reactions carried out using ACN as solvent conducted to the formation of benzaldehyde as major product (selectivity > 99%). Water and ACN are miscible in any proportion when pure,^44^ whereas a biphasic system is formed when salts^45^ or monosaccharides/disaccharides^46,47^ are added in a water/ACN mixture.

**Fig. 8:**
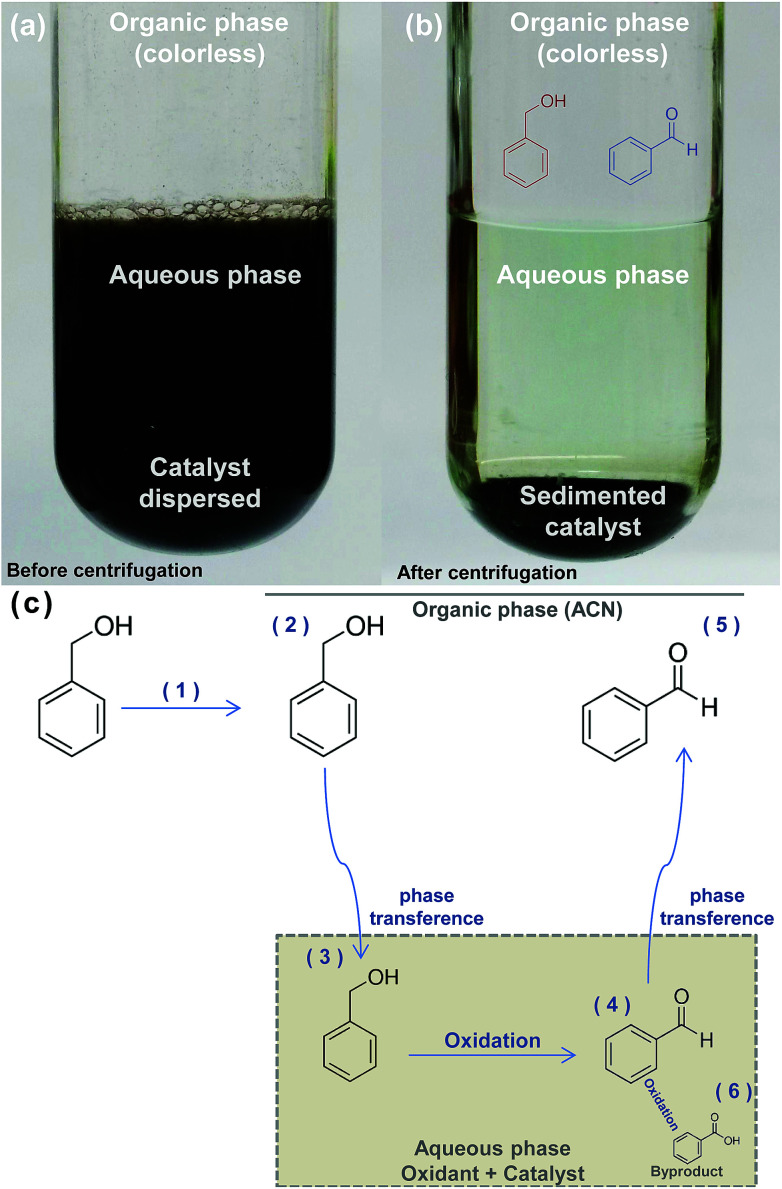
The pictures (a) and (b) show the reaction media before and after centrifugation for catalyst sedimentation, respectively. (c) The scheme shows the proposed reaction process for the oxidation of BnOH in the biphasic system: BnOH is introduced on ACN (1) and dissolved (2). With the addition of the oxidant and the formation of a biphasic system, fractions of BnOH are transfered to aqueous phase (3) where it is oxidized to BnCHO (4). The low aqueous solubility of BnCHO conducted to the diffusion of this specie to organic phase (5). Low concentration of BnCOOH are formed on aqueous phase (6) as a byproduct.

During the reactions, the nickel-based catalyst remains dispersed in the lower oxidant aqueous phase (Fig. 8(a)). BnOH conversion and BnCHO formation along the time was determined collecting a fraction of the superior organic phase. Table 2 summarizes the distribution of compounds (mmols) in both phases at the end of the reaction.

**Table 2:** BnOH, BnCHO and BnCOOH concentrations presents in organic (O) and aqueous (A) phases after reaction using Ni-based catalyst nanoparticles^a^^,^^b^

Phase^c^	BnOH mmols (×10^−3^)	BnCHO mmols (×10^−3^)	BnCOOH mmols (×10^−3^)
Organic	250	510	N.D.
Aqueous	2.0 (0.8%)^d^	1.7 (0.3%)^d^	4.2

a N.D. = not determined.

b Ni-doped glass annealed at 430 °C during 60 min under H_2_.

c After centrifugation, 3400 rpm, 6 min.

d The % of the species in aqueous phase related to organic phase.

According to Table 2, BnOH and BnCHO are predominantly present in organic phase in both reactions and only a small percentage of these products is present in aqueous phase, with a maximum value of 0.8% and 0.3% for BnOH and BnCHO, respectively. On the other hand, BnCOOH was only determined in the aqueous phase and in low concentration (4.2 × 10^−3^ mmol), which allowed us to infer that this compound is a byproduct. Thus, we have proposed a scheme for BnOH oxidation by NaOCl using the nickel-based catalyst (Fig. 8).

We propose the following steps for the reaction: BnOH is introduced in ACN and dissolved (Fig. 8(c) (steps 1 and 2)). The biphasic system is formed immediately when NaOCl 10–12% is introduced in the reaction. ACN forms the upper phase while the catalyst dispersed on the oxidant form the lower phase. The solubility of BnOH in water is ≈40 g L,^48^ allowing the phase transference from organic to aqueous phase (Fig. 8(c) (step 3)), where the oxidation occurs (Fig. 8(c) (step 4)). The solubility of BnCHO in water is ≈0.3 g L,^48^ inducing its phase transference to ACN (Fig. 8(c) (step 5)). The BnCHO diffusion from aqueous to ACN phase not only avoids a further oxidation, but also provides a high selectivity. In turn, BnCOOH is produced in the aqueous phase as a byproduct of the reaction (Fig. 8(c) (step 6)).

### Glass catalyst performance: Friedel–Crafts alkylation reaction of indole and benzaldehyde

Besides the investigation of the activity of nickel nanoparticles based catalyst for benzyl alcohol oxidation, its catalytic performance was tested for the Friedel–Crafts alkylation of indole 1 and benzaldehyde 2 for the synthesis of bis(indolyl)methane 3 under solvent-free conditions (Table 3). Initially, the reaction was carried out at 60 °C being the progress of reaction monitored by thin layer chromatography (TLC) analysis. After 3 h of reaction the total consumption of the indole 1 was observed and the desired product 3 was obtained with a yield of 90% (Table 3, entry 1). However, when the reaction time was reduced to 1.5 h no significant decrease in yield was observed (Table 3, entry 2). On the other hand, a temperature reduction provided the product 3 in only 35% of yield (Table 3, entry 3).

**Table 3:** Ni-based nano catalyzed Friedel–Crafts alkylation reaction of indole and benzaldehyde under solvent-free conditions^a^

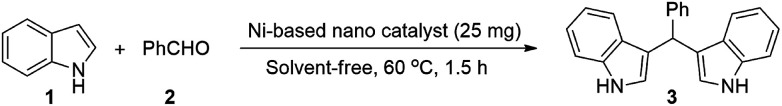				
Entry	Catalyst	*T* °C	Time h	Yield^b^ %
1	Ni-based^c^	60	3	90
**2**	**Ni-based** ^c^	**60**	**1.5**	**85**
3	Ni-based^c^	r.t.	1.5	35
4	Undoped	60	3	65
5	Unannealed	60	3	68
6	—	60	1.5	42
7	Silica glass	60	1.5	45

a Reaction conditions: indole (0.5 mmol), benzaldehyde (0.25 mmol) and catalyst (25 mg).

b Yield of pure product isolated by column chromatography and identified by ^1^H and ^13^C NMR.

c Nickel-doped germanophosphate glass annealed at 400 °C under H_2_(g) during 60 min. r.t. = room temperature (25 °C).

To examine the influence of Ni-based nanoparticles in the catalysis, the undoped germanophosphate glass and unannealed Ni-doped were evaluated. However, even after 3 h of reaction the starting materials were not consumed, providing the desired product in 65% and 68% yield, respectively (Table 3, entries 4 and 5). Furthermore, it is notable that in the absence of the catalyst and when silica glass was used, the product 3 was obtained in lower yield (Table 3, entries 6 and 7).

Having established a simple, robust and solvent-free methodology for the synthesis of bis(indolyl)methane 3 (Table 3, entry 2), it is interesting to note also the application of nickel species in this kind of catalysis, once it is rare in the literature.^49^ Additionally, the catalytic activity could be better for longer annealing times. As can be seen from SEM images (Fig. 4), the number of nickel-based nanoparticles became larger when the annealing time increases from 30 to 60 minutes. Furthermore, it is also possible to observe that with the increase of the annealing time the average size of the nanoparticles did not change considerably. This suggests that increasing the annealing time the catalytic activity will be enhanced since the number of particles will increase.

## Conclusions

In summary, germanophosphate glasses were developed for self-supported nickel-based nanoparticles synthesis onto glass surface by a bottom-up process. The nanoparticle growth is thermally dependent and favored under hydrogen atmosphere. Moreover, the annealing process under H_2_ enables the growth of nanoparticles with a reduced oxidation state covering homogeneously the glass surface. The catalytic performance of nickel-based nanoparticles was demonstrated for two different reactions. For benzyl alcohol oxidation, the catalyst shows high selectivity to benzaldehyde employing a strong oxidant and acetonitrile at mild conditions. Furthermore, the application of the catalyst has been successfully extended to Friedel–Crafts alkylation reaction of indole and benzaldehyde.

## Conflicts of interest

There are no conflicts to declare.

## Supplementary Material
